# Role of Neutrophil Extracellular Traps and Vesicles in Regulating Vascular Endothelial Permeability

**DOI:** 10.3389/fimmu.2019.01037

**Published:** 2019-05-09

**Authors:** Yonggang Ma, Xiaoyuan Yang, Victor Chatterjee, Jamie E. Meegan, Richard S. Beard Jr., Sarah Y. Yuan

**Affiliations:** ^1^Department of Molecular Pharmacology and Physiology, Morsani College of Medicine, University of South Florida, Tampa, FL, United States; ^2^Department of Biological Sciences, Biomolecular Research Center, Boise State University, Boise, ID, United States; ^3^Department of Surgery, Morsani College of Medicine, University of South Florida, Tampa, FL, United States

**Keywords:** cell-cell junction, endothelial barrier, extracellular vesicles, glycocalyx, inflammation, neutrophil extracellular traps, permeability

## Abstract

The microvascular endothelium serves as the major barrier that controls the transport of blood constituents across the vessel wall. Barrier leakage occurs during infection or sterile inflammation, allowing plasma fluid and cells to extravasate and accumulate in surrounding tissues, an important pathology underlying a variety of infectious diseases and immune disorders. The leak process is triggered and regulated by bidirectional communications between circulating cells and vascular cells at the blood-vessel interface. While the molecular mechanisms underlying this complex process remain incompletely understood, emerging evidence supports the roles of neutrophil-endothelium interaction and neutrophil-derived products, including neutrophil extracellular traps and vesicles, in the pathogenesis of vascular barrier injury. In this review, we summarize the current knowledge on neutrophil-induced changes in endothelial barrier structures, with a detailed presentation of recently characterized molecular pathways involved in the production and effects of neutrophil extracellular traps and extracellular vesicles. Additionally, we discuss the therapeutic implications of altering neutrophil interactions with the endothelial barrier in treating inflammatory diseases.

## Introduction

Serving as the blood-tissue interface, the vascular endothelial barrier plays a critical role in regulating host defense against infection or injury. Endothelial hyperpermeability is considered an important cause, as well as consequence, of inflammatory/immune responses associated with sepsis, trauma, ischemia-reperfusion injury, diabetes, and metastatic tumor development (1, 2). This pathological process involves complex cell-cell communications and molecular signaling. Among the multiple subtypes of leukocytes in the circulation, polymorphonuclear granulocytes (PMNs), or neutrophils, are the most impactful cells to vascular permeability, as they can alter endothelial barrier properties via direct contacts (adhesion and transmigration) and/or by secreting bioactive products capable of disrupting the barrier structure. Below, we discuss the effects of neutrophil-endothelium contact and neutrophil-derived factors on endothelial permeability.

## PMN-Endothelium Interactions

Neutrophils comprise the innate immune system providing the first line of defense against invading bacteria and acute injury (1). Traditionally, the life span of mature neutrophils is thought to be short, as they normally stay in the circulation for 5–10 h and subsequently infiltrate into tissues and die within the next 8–16 h (1, 2). Challenging this dogma, recent studies using *in vivo* labeling with ^2^H_2_O reveal that the life span of human circulating neutrophils lasts as long as 5.4 days (3), at least 10 times longer than previously reported. Another interesting finding is that after diapedesis, neutrophils can live in tissues for up to 7 days in the proinflammatory microenvironment (4). Whether and how neutrophil interaction with the microvascular endothelium affects their life span in the circulation, or in tissues, remain as a puzzle; however, evidence is accumulating that endothelial cells have the ability to educate neutrophils and modify their behavior during diapedesis (5, 6).

### Adhesion and Transendothelial Migration (TEM)

Neutrophil diapedesis is a tightly regulated process initiated with cell rolling along the microvascular (mainly venular) wall, followed by adhesion to endothelial surface and migration across the endothelium. The process is mediated by adhesion molecules whose expression is rapidly upregulated by inflammatory cytokines, including tumor necrosis factor (TNF)-α and interleukin (IL)-1β. In particular, ligation of neutrophil P-selectin and endothelial E-selectin slows down neutrophils and enables their rolling under relatively high shear stress (7, 8). Subsequently, firm adhesion is secured via the binding of neutrophil CD11/CD18 integrins to endothelial adhesion molecules (7, 9). Transmigration occurs through the para-cellular route via endothelial cell-cell junctions (6), or through the transcellular route across endothelial cell body (10); the former is considered the predominant pathway (~70–90%) (11). In 2004, Carman and colleagues identified microvilli-like projections on endothelial cell surface that form “transmigratory cup” to provide directional guidance for leukocyte trafficking.

### Reverse TEM (RTEM)

To prevent excessive inflammation and secondary tissue injury, activated neutrophils at sites of inflamed tissue have to be timely cleared, which can happen in several ways (12). Apoptosis and subsequent clearance by macrophage phagocytosis are thought to be a common fate to innate immune cells, such as neutrophils, eosinophils, and basophils (12–14). However, a growing body of evidence suggests that neutrophils can re-enter the circulation through RTEM (15–17). Some mechanisms have already been revealed. For instance, leukotriene (LT)B4 can disrupt the junctional adhesion molecule-C and facilitate neutrophil reverse migration (18). Macrophages are shown to promote reverse migration through neutrophil redox-Src family kinase signaling, whereas Src deficiency impairs neutrophil RTEM (19). This might represent another mechanism of macrophage clearance of neutrophils, in addition to macrophage phagocytosis of apoptotic neutrophils. Interestingly, reverse transmigrated neutrophils display high expression of intercellular adhesion molecule (ICAM)-1, which is minimally expressed in circulatory neutrophils (20); the functional implication of this phenotype change is unclear. It is suggested that RTEM assists in the dissemination of systemic inflammation (18). Therefore, neutrophil RTEM contributes to not only resolution, but also propagation, of inflammation. More work is warranted to establish the pathophysiological significance of neutrophil TEM/RTEM. Of particular interest is how these processes affect endothelial barrier property.

## Endothelial Barrier

**Figure 1:**
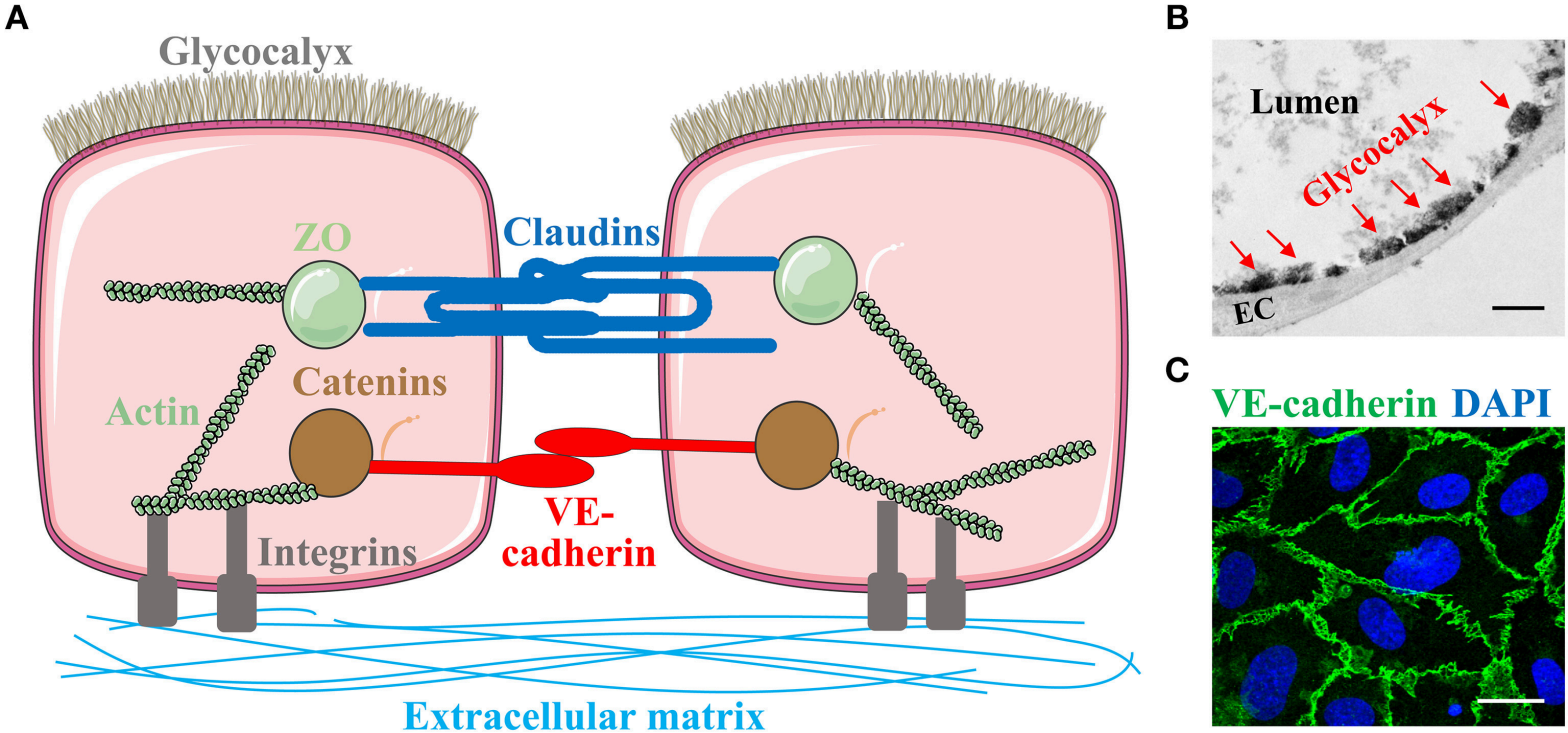
Endothelial barrier structure**. (A)** The endothelial barrier of exchange microvessels is composed of endothelial cells connected to each other via junctions, with its luminal surface protected by glycocalyx and basolateral side anchored to the extracellular matrix in the basement membrane through focal adhesions. Endothelial cell-cell adhesion is mediated by two types of junction: the claudin-based tight junction which is linked to the actin cytoskeleton through zonula occludens (ZO), and the VE-cadherin-based adherens junction which binds actin through catenins. Some images of cells or organelles were obtained from Servier Medical Art (www.servier.com). **(B)** Glycocalyx in mouse lung capillary under transmission electron microscopy. EC, endothelial cells. Red arrows indicate glycocalyx. Scale bar = 1 μm. **(C)** Immunofluorescent staining of VE-cadherin on human umbilical vein endothelial cells. Green, VE-cadherin. Blue, DAPI. Scale bar = 20 μm.

The endothelial barrier of exchange microvessels (capillaries and post-capillary venules) has three major components (Figure 1A): cell-cell junctions, luminal surface glycocalyx, and basolateral focal adhesions (9). These components act in concert to determine the barrier permeability.

### Cell-Cell Junctions

In the microvasculature, at least two types of junctions are identified: tight junctions (TJs) and adherens junctions (AJs) (Figures 1A,C). TJs have been extensively studied with respect to blood-brain barrier (BBB) and blood-retinal barrier due to their predominant expression in cerebral and retinal microvasculature, respectively. TJs are composed of occludins and claudins, tetraspanning molecules linked to the actin cytoskeleton through cytoplasmic adaptor proteins, zonula occludens (ZO) (21). AJs are considered the primary junctions in the peripheral microvasculature. They mainly consist of the transmembrane homophilic dimers, vascular endothelial (VE)-cadherin, which are anchored to the actin cytoskeleton through catenins (α-, β-, γ-, and p120-catenin) (22, 23).

Disassembly or opening of AJs can lead to increased paracellular permeability (24). Given the rapid nature of leak responses to inflammatory agonists, such as histamine and vascular endothelial growth factor (VEGF), which occur in minutes following stimulation (25), dynamic changes of barrier conformation via post-translational modification (PTM) of junction molecules are considered to be an important underlying mechanism. Protein phosphorylation is a commonly studied PTM. It is generally accepted that tyrosine phosphorylation of VE-cadherin triggers its dissociation from catenins, thereby weakening the junction anchorage to cytoskeleton (26, 27). VE-cadherin can be phosphorylated by tyrosine kinase Src or protein kinase C (PKC) (28). Phosphatases also regulate VE-cadherin de-phosphorylation thereby altering barrier function (27). There is evidence that VE-cadherin phosphorylation and dephosphorylation at different sites differentially regulate vascular permeability (29). Other junction proteins, such as β-catenin, can be phosphorylated by proline-rich tyrosine kinase (Pyk)-2 (26), promoting its dissociation from the VE-cadherin junction (30). In addition to junction molecules, cytoskeleton molecules undergo conformational changes upon phosphorylation. For example, myosin light chain kinase (MLCK) phosphorylates myosin light chain and triggers actin-myosin contraction, pulling away the neighboring cells and leading to intercellular gaps (31).

Additional PTM mechanisms implicated in junction permeability include nitrosylation (32) and lipidation. We have recently identified a new lipidation pathway, endothelial protein palmitoylation mediated by palmitoyl acyltransferase DHHC21, in promoting neutrophil-endothelium adhesion and microvascular permeability (33).

### Glycocalyx

Covering the luminal surface of endothelial barrier is a matrix meshwork, glycocalyx, constituted with glycosaminoglycans (GAG), proteoglycans, and glycoproteins (Figures 1A,B) (34). The GAG chains contain heparan sulfate, chondroitin sulfate, and hyaluronic acid; the latter binds to a transmembrane glycoprotein, CD44 (35). Proteoglycans, the core transmembrane proteins, include syndecans and glypicans. Glycoproteins include selectins and integrins, which participate in neutrophil adhesion and other intravascular processes such as coagulation and fibrinolysis (36). A dynamic equilibrium exists between the biosynthesis and shedding of endothelial glycocalyx constituents, which determines glycocalyx thickness, morphology, and function (37).

An important function of endothelial surface glycocalyx is providing a protective layer to prevent the endothelium from being exposed to circulating cells or agents (38). It participates in a number of biological events, including neutrophil-endothelium cross-talk (36, 37). Glycocalyx disruption contributes to compromised endothelial barrier integrity and increased microvascular permeability (39). Shedding of glycocalyx constituents occurs via enzymatic digestion by metalloproteases and hyaluronidase, or non-enzymatic stimulation such as oxidative stress (40, 41); both are activated during neutrophil-mediated innate immune response. Our recent study revealed an important role of a disintegrin and metalloproteinase 15 (ADAM15) in glycocalyx destruction (35). In particular, ADAM15 is upregulated during infection, and it cleaves glycocalyx constituents, including CD44. The cleaved products target endothelial cells in a paracrine manner inducing barrier dysfunction and microvascular leakage (35). We also show that syndecan-3/4 can be cleaved by thrombin to produce ectodomain fragments, and these fragments trigger AJ disorganization and stress fiber formation, causing elevated para-cellular permeability (42). Consistent with our findings, other studies show that in septic lungs, glycocalyx degradation leads to increased availability of endothelial surface receptors for neutrophil adhesion molecules and thereby facilitating neutrophil infiltration (43).

### Focal Adhesions

At the basolateral side, endothelial cells are attached to extracellular matrix (ECM) through focal adhesions, complex transmembrane structures consisting of integrins, focal adhesion kinase (FAK), and adaptor proteins (44). While they are essential to the maintenance of endothelial barrier properties under basal conditions (28, 31), their activation or redistribution contributes to paracellular leakage (45). Studies have shown that both FAK and β1/3 integrins are required for microvascular leak responses to blood clot fibrinolysis products (46).

FAK is a non-receptor tyrosine kinase that controls focal adhesion assembly and distribution. We have previously reported that FAK mediates endothelial barrier dysfunction caused by C5a-activated neutrophils, an effect dependent on FAK signaling activity (9). Certain inflammatory mediators secreted by neutrophils can activate FAK by inducing its phosphorylation (47, 48). FAK phosphorylation at tyrosine-925 exposes the SH2-binding site for Grb2, which triggers downstream signals involving Ras-ERK1/2 and MLCK-dependent actomyosin contraction (9). FAK inhibition alleviates venular hyperpermeability caused by neutrophils or VEGF (48, 49).

## PMN Regulation of Endotehlial Permeability

**Figure 2:**
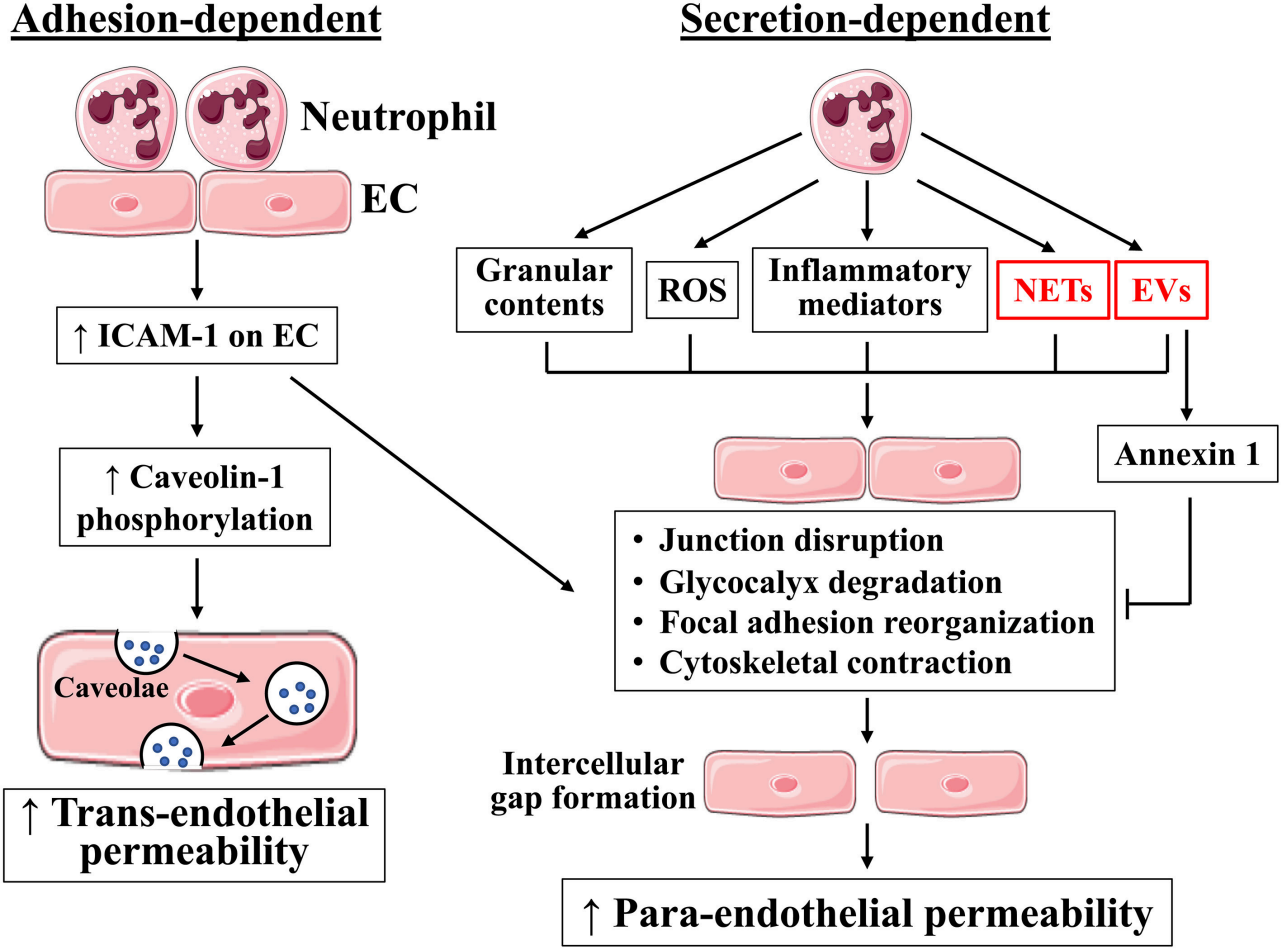
Neutrophils regulate endothelial barrier function through adhesion-dependent and secretion-dependent mechanisms. Neutrophil adhesion to endothelial cells activates ICAM-1 signaling, which increases permeability through both para-endothelial and trans-endothelial routes. In addition, neutrophils can generate ROS, inflammatory mediators, granular contents, neutrophil extracellular traps (NETs), and extracellular vesicles (EVs), which in turn cause junction disruption, glycocalyx degradation, focal adhesion reorganization, and cytoskeletal contraction, leading to intercellular gap formation and increased para-endothelial permeability. Neutrophils also release barrier-protecting factors, including annexin 1. EC, endothelial cells. Blue dots, blood constituents. Images of cells were obtained from Servier Medical Art (www.servier.com).

Neutrophils regulate endothelial permeability by altering the structure and function of the aforementioned barrier components: cell-cell junctions, glycocalyx, and focal adhesions. During inflammation, activated neutrophils exert detrimental effects to these structures via direct contacts established during adhesion and transmigration, or via secretion of barrier-disrupting molecules (Figure 2). Neutrophil respiratory burst produces reactive oxygen species (ROS), and neutrophil degranulation produces myeloperoxidase, elastase, cathepsin G, and metalloproteases; all are capable of cleaving glycocalyx. Glycocalyx injury results in the loss of protective layer and exposure of endothelial surface receptors for neutrophil adhesion, further activating neutrophil-endothelium interactions. In endothelial cell-cell junctions, VE-cadherin is particularly susceptible to enzymatic degradation, and its cleavage by metalloproteases, elastase, and cathepsin G leads to impaired junction integrity (27, 50). At the basal lateral site, FAK activation and integrin engagement in response to neutrophil TEM, or their secreted products, promote focal adhesion assembly and redistribution in alignment with contractile cytoskeleton, providing support for endothelial cells to undergo conformational changes. Below we discuss further details on how neutrophils regulate endothelial barrier function, focusing on adhesion-dependent and secretion-dependent pathways.

### PMN Adhesion and Endothelial Permeability

Traditionally, neutrophil adhesion followed by TEM is thought to physically damage the endothelial barrier. Challenging this dogma, transmission electron microscopic studies show that no tracer leakage is coupled with neutrophil TEM, and that TEM can occur without impairing the junctional structure (9, 51). While the physical attachment of neutrophils to the endothelium can exacerbate barrier injury, it is not required for hyperpermeability responses.

Several studies suggest the importance of adhesion molecule engagement in barrier regulation. In particular, ICAM-1 engagement in the absence of leukocytes is sufficient to increase endothelial permeability (52). Ligation of endothelial ICAM-1 can directly increase permeability (26, 52), and antibodies blocking ICAM-1 alleviate endothelial injury during inflammation (53). The signal transduction downstream from ICAM-1 engagement involves Rac, which subsequently activates NADPH oxidases and causes ROS production. ROS activate Src or Pyk2, which phosphorylate VE-cadherin molecules and promote their dissociation. Src also activates FAK and initiates focal adhesion redistribution. Moreover, ICAM-1 ligation induces dissociation of vascular endothelial protein tyrosine phosphatase from VE-cadherin, promoting VE-cadherin phosphorylation (5). In the cytoskeleton, MLCK is also activated after ICAM-1 ligation (54). Together, these signaling reactions lead to a focal adhesion-supported cytoskeleton contraction and junction opening (9, 55).

In addition to its role in paracellular permeability, neutrophil adhesion also activates the trans-endothelial route of protein transport through ICAM-1 signaling (Figure 2) (53). ICAM-1 ligation facilitates Src phosphorylation of caveolin-1, a major component of caveolae. Caveolae serve as the primary mechanism of moving albumin across the endothelial cell body, from the luminal side of cell membrane toward basement membrane. The relative contributions of ICAM-1 to paracellular vs. transcellular permeability remains to be established.

### PMN Secretion and Endothelial Permeability

It is well-known that neutrophils cause barrier dysfunction by producing ROS, secreting inflammatory mediators, and releasing granular contents (Figure 2) (1, 56). The mechanisms by which ROS induce vascular hyperpermeability include junction disruption and endothelial cell contraction mediated by MLCK, MAPK, PKC, tyrosine kinases, and Rho GTPases (56, 57). Other permeability-increasing agents released by neutrophils include TNF-α, IL-1β, and chemokines CXCL1, 2, 3, and 8 (5, 58). Through degranulation, neutrophils release metalloproteases, elastase, cathepsin G, and proteinase 3; these proteolytically active enzymes can breakdown junctional complexes, glycocalyx constituents, and focal adhesion components (59, 60). Additionally, neutrophil-derived LTA4 induces the synthesis of biologically active LTB4 (61), LTB4 then further activates neutrophils to release heparin-binding protein (a granule component), causing endothelial cell contraction (62). TNF-α can stimulate neutrophil release of elastase and cathepsin G, which cleave VE-cadherin and disrupt the junction integrity (63). These findings suggest a synergistic action of neutrophil-derived agents in regulating barrier property. While these mechanisms represent the traditional pathways of neutrophil secretion-induced permeability, below we discuss two newly characterized barrier-altering factors produced by neutrophils.

#### Neutrophil Extracellular Traps (NETs)

Neutrophils can release nuclear components (DNA and histones) and cytoplasmic granular proteins (elastase, myeloperoxidase, cathepsin G, and metalloproteases) into the extracellular environment, which form NETs to trap invading microorganisms. This pathogen-killing mechanism was first described by Brinkmann et al in 2004 (64). Peptidylarginine deiminase (PAD)4 plays a key role in NET formation by converting arginyl residues on chromatin histones to citrulline (which lacks positive charge), releasing the ionic bonds that constrain nuclear DNA to nucleosomes and thus freeing the strands of DNA to unfurl (65). In parallel, neutrophil elastase translocates to the nucleus and degrade histones, facilitating chromatin decondensation (66). Subsequently, decondensed chromatin fused with granule components is released to extracellular space.

NETs are originally thought to be generated by neutrophils undergoing cell death, a process known as suicidal NETosis. Non-suicidal vital NETosis was subsequently described, which occurs via blebbing of the nuclear envelope and vesicular exportation, thus displaying intact plasma membrane and viable neutrophils (67, 68). Additionally, Yousefi et al. identified mitochondrial NET, which is formed in living neutrophils and contains mitochondrial, but not nuclear, DNA (69). It is unclear whether and how these different types of NETs coexist, and what distinct functions they exert.

NET production can be induced by biological and chemical agents, including live bacteria, ROS, inflammatory cytokines (e.g., IL-1β, IL-8, and TNF-α), phorbol 12-myristate 13-acetate (PMA), and calcium ionomycin (70). NET formation is enhanced in infection and inflammation-associated diseases. The primary function of NETs is to trap pathogens and prevent dissemination of infection, being protective (64). Subsequently, NETs have been shown to be detrimental in multiple diseases including lung disease, thrombosis, cancer, and autoimmune disease (71). For instance, NETs exhibit the pro-inflammatory feature in chronic airway disease (72). The finding that PAD4 inhibition decreases arterial thrombosis in apolipoprotein-E^−/−^ mice indicates the pro-coagulant nature of NETs (73). NETs are recently shown to awaken dormant cancer cells and facilitate tumor metastasis through the activation of integrin and FAK/ERK/MLCK/YAP signaling by laminin fragments generated by neutrophil elastase and MMP-9 cleavage (74). NETs associated components are potential inducers of autoantibody production, a hallmark for auto-immune diseases. Not surprisingly, blocking NET formation decreases disease severity in a mouse model of systemic lupus erythematosus (75). A recent study demonstrates that partial PAD4 deficiency (PAD4^+/−^ or DNase I treatment) reduces lung injury and improves survival in a murine model of bacterial pneumonia, while PAD4^−/−^ mice show increased bacterial load and inflammation (76). This finding highlights the pleiotropic roles of NETs in pro-inflammation and anti-inflammation, which needs to be taken into consideration when targeting NETs as therapeutics.

**Figure 3:**
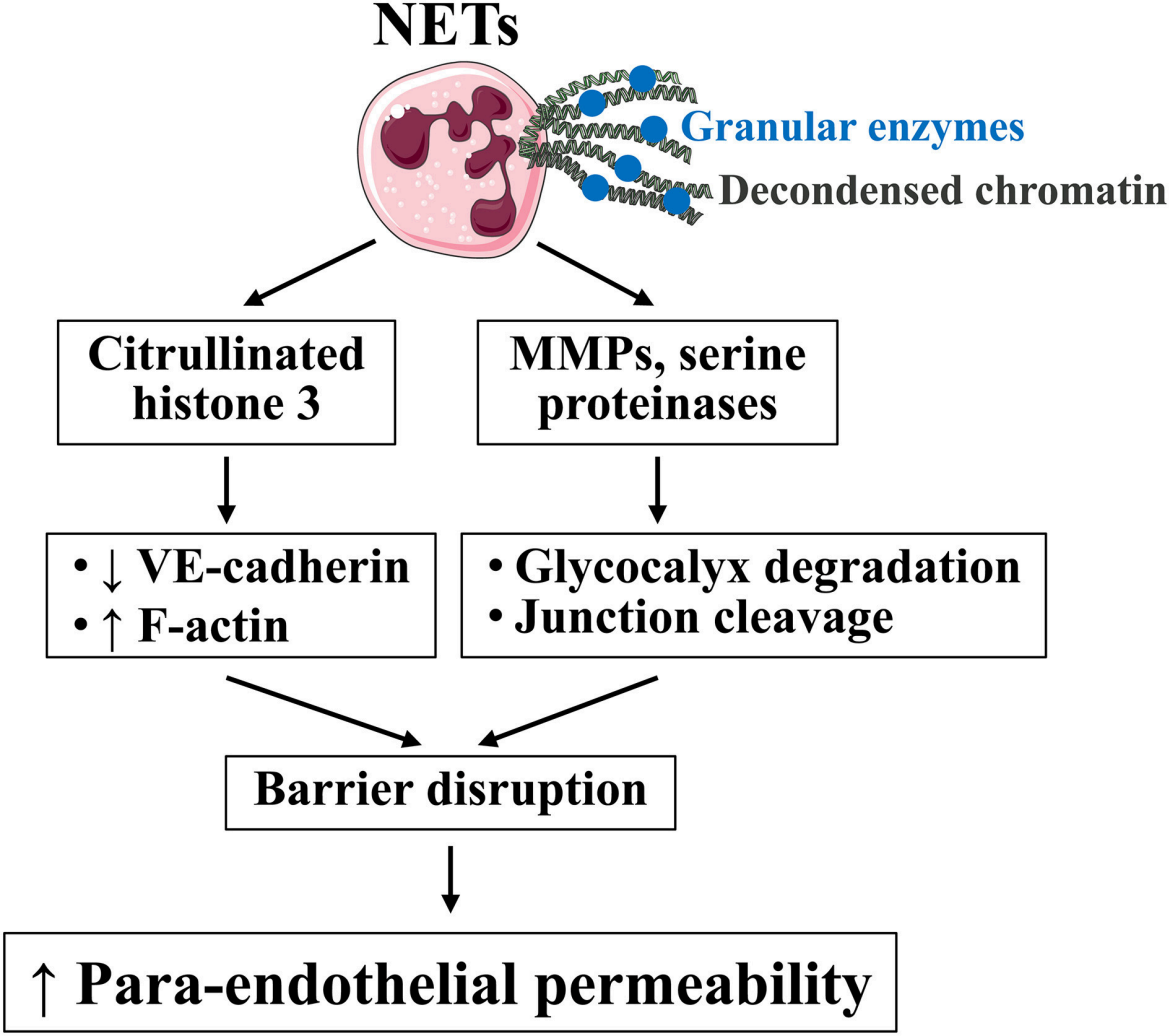
Effects of specific NET constituents on endothelial barrier function. NETs are composed of decondensed chromatin (e.g., citrullinated histone 3) and granular enzymes (MMPs and serine proteinases). Citrullinated histone 3 induces actin stress fiber formation and VE-cadherin junction discontinuity; MMPs and serine proteinases cleave glycocalyx and other barrier molecules; both lead to increased para-endothelial permeability. Images of cells were obtained from Servier Medical Art (www.servier.com).

With respect to NET regulation of microvascular permeability, we are at the very early stage of understanding NET's effects on endothelial barriers and their underlying mechanisms. *In vitro*, NETs increase the flux of albumin or 10-kDa dextran across endothelial cell monolayers (77, 78). Neutralizing NET components by DNase 1, or inhibition of NET formation by PAD2/4 inhibitor or PAD4 gene deletion, reduces lung vascular permeability in murine models of transfusion-related acute lung injury and LPS-induced endotoxemia, respectively (76–78). Our recent study reveals that citrullinated histone 3, a major protein component of NETs, causes microvascular leakage and barrier dysfunction by disrupting AJs and rearranging contractile cytoskeleton in endothelial cells (79). Consistent with our finding, others show that NETs increase albumin permeability through disrupted AJs (80). Serine proteinases (e.g., neutrophil elastase) and MMPs, enriched in NETs, can cleave VE-cadherin and compromise junction integrity (63, 81). MMPs further activate barrier-disrupting cytokines and chemokines, such as IL-1β, TNF-α, and CXCL8 (82, 83), which may amplify the hyperpermeability signaling. Figure 3 depicts effects of specific NET components on endothelial permeability.

The NET pathway is also under negative regulation. Lactoferrin, an iron-binding protein present in secondary granules of neutrophils, is released during neutrophil degranulation. A recent study by Okubo and colleagues demonstrates that lactoferrin suppresses NET formation, acting as an intrinsic inhibitor of NETs (84). Mice deficient in the lipoxin receptor 2 generate more NETs, leading to elevated lung injury and mortality after pneumonia (76). Thus, lipoxin receptor 2 may negatively regulate NET formation. Additionally, activated protein C (APC), a natural anticoagulant, is known to protect barrier function and decrease vascular permeability. Recent evidence shows that APC inhibits NET formation (85). It would be interesting to investigate whether NET inhibition by APC contributes to its barrier-protective effects.

#### Neutrophil-Derived Extracellular Vesicles

Extracellular vesicles (EVs) are heterogeneous membrane enveloped structures released by a variety of cells into body fluids (86). Based on their size and formation pathways, EVs are divided into 3 types: apoptotic bodies, microparticles (also known as microvesicles), and exosomes. Apoptotic bodies are the largest EVs with a diameter of 800 to 5,000 nm. They are released during the last stage of apoptosis, characterized by a permeable plasma membrane with externalized phosphatidylserine. This process is mediated by caspase and Rho-associated kinase I. Microparticles, ranging 100–1000 nm in diameter, are formed by the outward blebbing of the cell membrane, a process called “ectocytosis.” During its formation, the cytoskeleton is reorganized, and phosphatidylserine is redistributed to the outside of the plasma membrane, which involves multiple complex pathways, including calcium signaling and Rho-associated kinase I and II, nuclear factor-κB, p38MAPK, or TNF-related apoptosis-inducing ligand (87). Exosomes are the smallest EVs of 30–120 nm in diameter and exhibit a cup-like shape (88). In contrast to apoptotic bodies and microparticles that derive from plasma membrane, exosomes stem from the endosomal system. Exosomes are intraluminal vesicles contained in multivesicular bodies, which then fuse with the plasma membrane and are released into extracellular environment (89). The formation and secretion of exosomes are regulated by endosomal sorting complexes required for transport-dependent (90) and -independent manners (tetraspanins, lipid rafts, and Rab GTPases) (86, 91).

Originally thought as inactive cell debris, EVs have been studied as biomarkers for cell injury. Recently, growing evidence has emerged showing that EVs are active players in cell-cell communication (86, 89). EVs carry nucleic acids (e.g., microRNA), peptides, proteins, carbohydrates, and lipids that act as bioactive molecules to regulate multiple biological processes, including angiogenesis, immune response, cell migration and differentiation. In addition, EV cargos can be exchanged between cells as a mean of cell-cell communication or recycling.

EVs in the blood mainly derive from blood cells (leukocytes, platelets) and vascular cells (endothelial cells) (86, 92); their molecular property and cargo contents vary depending on the origin and pathophysiological state of parent cells that produce them. Neutrophil-derived EVs are in small amounts under normal conditions, but significantly increased in the blood during sepsis and inflammation (93–95). Pathophysiological stimuli, such as bacteria, complements, inflammatory cytokines, calcium, and platelet activating factor, are able to induce neutrophil production of EVs (95). In general, these EVs are considered to be foe to the endothelial barrier because they contain pro-inflammatory cargo, although the distinct effects of specific cargo contents remain to be established.

**Figure 4:**
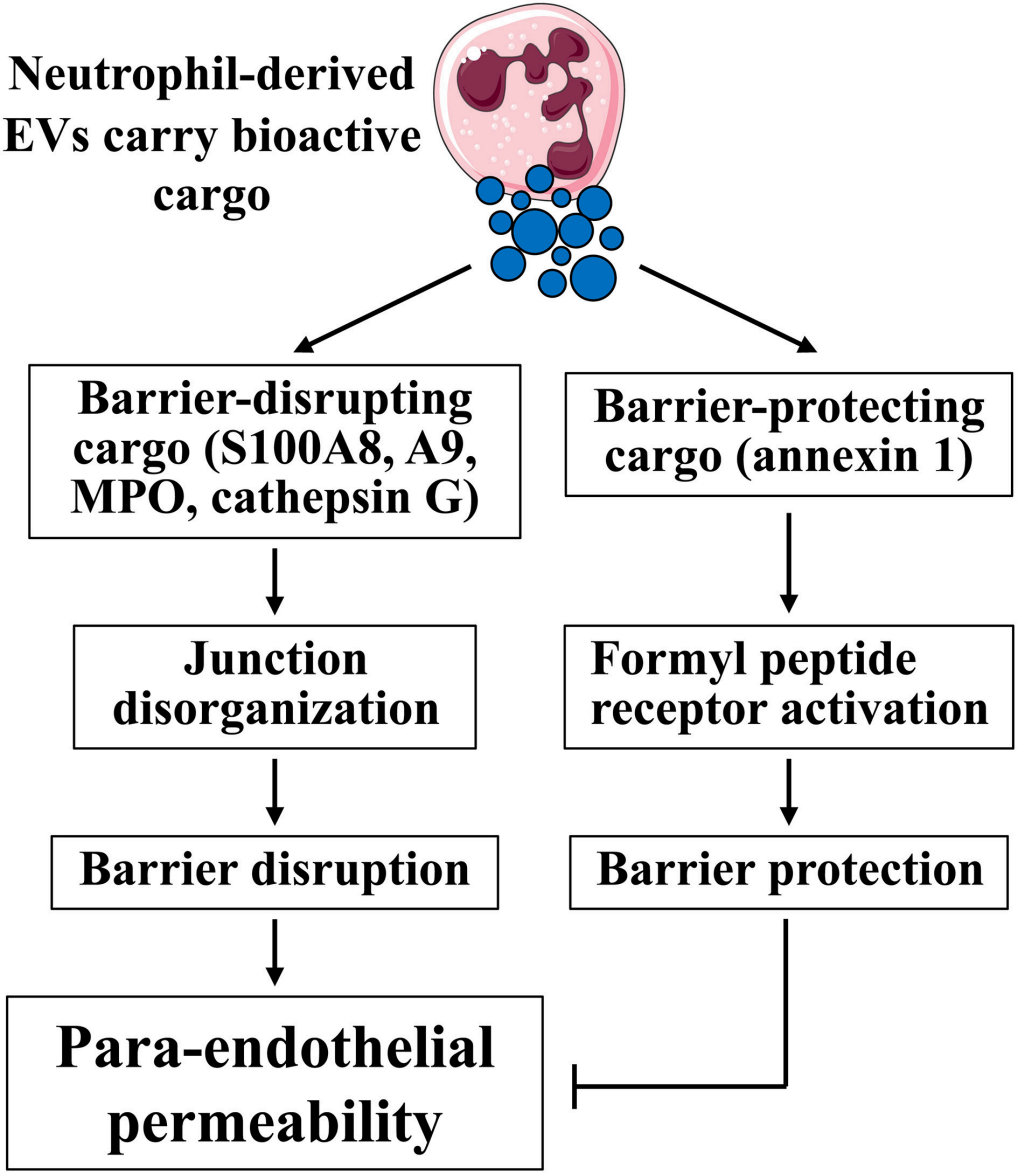
Effects of neutrophil-derived EVs on endothelial barrier function. Neutrophil-derived EVs display either positive or negative impact on endothelial permeability depending on their cargo contents. Barrier-disrupting cargo, such as S100A8, A9, MPO, and cathepsin G, are able to disrupt junction integrity and increase permeability. In contrast, barrier-protecting cargo, such as annexin 1, maintain junction integrity, and decrease permeability. Images of cells were obtained from Servier Medical Art (www.servier.com).

We propose that neutrophil-derived EVs possess both barrier-disrupting and barrier-protecting capabilities depending on their cargo components (Figure 4). In particular, proteomic analysis reveals that microparticles generated from fMLP-activated neutrophils contain > 300 different proteins, including pro-inflammatory S100A8, S100A9, MPO, and cathepsin G (96). S100A8, S100A9, or S100A8/A9 complexes induce F-actin and ZO-1 disassembly; they also increase endothelial monolayer permeability through activating p38 and ERK1/2 signaling pathways via binding to receptors TLR4 and RAGE (97). MPO can bind to the glycocalyx heparan sulfate by ionic interaction (independent of its catalytic property), which induces the release of neutrophil granular proteinases to cause syndecan-1 shedding and glycocalyx impairment (98). Cathepsin G is known to increase endothelial permeability to albumin through the detachment of plasminogen activator inhibitor-1 from the subendothelial matrix, causing F-actin rearrangement (99). In addition, cathepsin G can degrade VE-cadherin and impair junction integrity (100). All these studies suggest that neutrophil-derived EVs may have permeability-enhancing effects.

Other studies suggest the beneficial role of EVs in vascular homeostasis and endothelial permeability (101–104). For example, neutrophils adhering to endothelial cells generate microparticles enriched of annexin 1, an anti-inflammatory and pro-resolving protein that inhibits neutrophil adhesion and recruitment (104). Annexin 1, also known as annexin A1 or lipocortin 1, is expressed by brain microvascular endothelial cells and mediates the anti-inflammatory effects of glucocorticoid hormones. Annexin 1^−/−^ mice display increased BBB permeability as a result of disrupted TJs and disorganized actin cytoskeleton, which could be rescued by exogenous annexin 1 administration (105). This indicates that annexin 1 maintains endothelial tight junctions and BBB homeostasis (Figure 4). Annexin 1 is also shown to prevent inflammation-induced impairment in cerebrovascular endothelial barrier function (106). Annexin 1 targets endothelial cells by binding to G protein-coupled receptor formyl peptide receptor (or lipoxin A4 receptor) and subsequently activating intracellular signaling (107, 108). Therefore, the net effect of neutrophil-derived EVs may depend on their cargo contents and balance between barrier-protecting and barrier-disrupting molecules.

## Therapeutic Implications

Vascular leakage is a common complication of various infectious or inflammatory diseases (109). The importance of protecting endothelial barriers and repairing leaky vessels has increasingly been appreciated, as evidenced by many recent trials aimed at targeting endothelial dysfunction. Molecules that demonstrate the capability to enhance barrier property include sphingosine 1-phosphate, APC, angiopoietins, PKC inhibitors, RhoA inhibitors, corticosteroids, histamine receptor blockades, anti-VEGF, and vasopressin type 1a agonists (110–113). While all of them display beneficial roles in animal models, many do not demonstrate clinical efficacy. For example, anti-neutrophil adhesion therapies using monoclonal antibodies against CD18 or ICAM-1 have failed to improve clinical outcomes in patients with burn injury, traumatic shock, and ischemia-reperfusion injury (114). The lack of endothelial barrier-specific therapies highlights the need for further studies to identify novel therapeutic targets. As accumulating evidence supports the important contribution of NETs and neutrophil-derived EVs to vascular barrier dysfunction, additional work is warranted to investigate whether altering their production, or interfering their mechanistic pathways, has clinical implications or therapeutic potential. In view of the complexity of human diseases, targeting specific molecular pathways key to barrier structure and function holds great promise to the treatment of diseases associated with aberrant immune/inflammatory response.

## Author Contributions

All authors have made a substantial and intellectual contribution to this work and approved it for publication.

### Conflict of Interest Statement

The authors declare that the research was conducted in the absence of any commercial or financial relationships that could be construed as a potential conflict of interest.
